# C-Reactive Protein, Interleukin 6 and Lung Cancer Risk: A Meta-Analysis

**DOI:** 10.1371/journal.pone.0043075

**Published:** 2012-08-17

**Authors:** Bo Zhou, Jing Liu, Ze-Mu Wang, Tao Xi

**Affiliations:** 1 School of Life Science and Technology, China Pharmaceutical University, Nanjing, China; 2 Jiangsu Center of Safety Evaluation for Drugs, School of Pharmaceutical Sciences, Nanjing University of Technology, Nanjing, China; 3 Department of Cardiology, The First Affiliated Hospital of Nanjing Medical University, Nanjing, China; Cedars-Sinai Medical Center, United States of America

## Abstract

**Purpose:**

Epidemiologic findings are inconsistent concerning the associations between C-reactive protein (CRP), interleukin 6 (IL-6) and lung cancer risk. We conducted a meta-analysis of epidemiologic studies to examine these associations.

**Methods:**

A systematic literature search up to October 2011 was performed in MEDLINE and EMBASE. Study-specific risk estimates were pooled using a random-effects model.

**Results:**

The 10 studies on CRP involved a total of 1918 lung cancer cases. The pooled RR of lung cancer for one unit change in natural logarithm (ln) CRP was 1.28 (95% CI 1.17–1.41). There was no statistically significant heterogeneity among studies (*P* = 0.116; I^2^ = 36.6%). We also found that CRP was significantly associated with increased risk of lung cancer among men (RR 1.18, 95% CI 1.09–1.28) but not among women. The 5 studies on IL-6 involved a total of 924 lung cancer cases. The pooled RR of lung cancer for one unit change in ln IL-6 was 1.28 (95% CI 0.92–1.79), however, statistically significant heterogeneity was found. After excluding the study contributing most to the heterogeneity, the summary estimate was essentially unchanged.

**Conclusion:**

CRP was associated with increased risk of lung cancer, especially among men. There was no significant association between IL-6 and lung cancer risk.

## Introduction

According to the International Agency for Research on Cancer for 2008, about 1.6 million individuals were diagnosed with lung cancer and 1.4 million died as a result, which makes it the first-leading cause of cancer-related deaths in men and second in women [1]. In the United States, the overall five-year survival rate for patient with lung cancer is 16%, but for patients diagnosed with localized disease is up to 53% [2]. Thus, early diagnosis remains a key factor for improving the poor survival rate.

Even though smoking is the major cause, most smokers (>80%) never develop lung cancer, suggesting that additional cofactors are also required [3]. There is growing evidence that chronic pulmonary inflammation plays an important role in lung cancer development [4], [5]. Moreover, inflammatory lung conditions such as chronic obstructive pulmonary disease, chronic diffuse infiltrative lung diseases, and polymorphisms in inflammation-related genes have all been linked with increased lung cancer risk [6], [7].

C-reactive protein (CRP), a systemic marker of chronic inflammation, is an acute phase reactant protein that increases during the host response to tissue injury, including infection, trauma, myocardial infarction, surgery, and cancer [8]. Some studies have shown that serum CRP levels are associated with risk of cardiovascular disease [9]. In recent years, elevated levels of CRP have been reported as a risk factor for the development of colon cancer [10]. Interleukin 6 (IL-6), a major proinflammatory cytokine, is produced in a variety of tissues including activated leukocytes, adipocytes, and endothelial cells. It is suggested that circulating IL-6 may be associated with lung cancer because it is expressed in premalignant epithelial cells, and the expression is associated with a poor prognosis in lung cancer patients [11].

During the last decade, several epidemiologic studies have evaluated the associations between CRP, IL-6 and lung cancer risk. A meta-analysis published in 2009 found that a natural log (ln) unit increase in CRP was associated with a 1.32-fold increase (95% confidence interval [CI] 1.08–1.61) in lung cancer risk, however, significant heterogeneity was found. And this estimate was based on only 551 lung cancer cases. The pooled effect estimate based on only 131 cases suggested that IL-6 was not associated with lung cancer risk (relative risk [RR] 1.05, 95% CI 0.72–1.52) [12]. Subsequently, several epidemiologic studies with large sample sizes or long-term follow-up have been performed regarding CRP, IL-6 and the risk of lung cancer. Therefore, we undertook this meta-analysis to further clarify these associations.

## Materials and Methods

### Search Strategy

A systematic literature search up to October of 2011 was performed in MEDLINE and EMBASE to identify relevant studies. Search terms included “C-reactive protein” or “Interleukin 6′′ combined with “lung cancer” “lung carcinoma” or “lung neoplasm”. No language restrictions were imposed. The titles and abstracts were scanned to exclude any clearly irrelevant studies. The full texts of the remaining articles were read to determine whether they contained information on the topic of interest. Furthermore, to find any additional published studies, a manual search was performed by checking all the references of retrieved articles. All searches were conducted independently by 2 authors (BZ and JL). The results were compared, and any questions or discrepancies were resolved through iteration and consensus.

### Study Selection

To be eligible, studies had to fulfill the following 4 inclusion criteria: 1) prospective or case-control study design; 2) report results on blood CRP or IL-6 levels; 3) lung cancer incidence as the outcome of interest; and 4) reported RR (or odds ratio [OR] estimates in case-control studies) or hazard ratios (HR) estimates with their corresponding 95% CI (or sufficient data to calculate of these effect measure).

### Data Extraction

Information from studies was extracted independently by 2 researchers (BZ and JL), with disagreements resolved by consensus. The following data were collected: the first author’s last name, year of publication, study population, country in which the study was performed, study design, study participants age range, sample size (cases and controls or cohort size), measure and range of exposure, and RR estimates with corresponding 95% CIs for CRP or IL-6 levels. If a study provided several risk estimates, the most completely adjusted estimate was extracted.

### Statistical Analysis

Study-specific risk estimates were extracted from each article, and log risk estimates were weighted by the inverse of their variances to obtain a pooled risk estimate. Studies were combined by using the DerSimonian and Laird random-effects model, which considers both within- and between-study variations [13]. The primary analyses combined ln RR associated with one unit change in ln CRP (mg/l) or ln IL-6 (pg/ml). Several studies did not report a risk estimate for one unit change in ln CRP or ln IL-6. For these studies, we used the method proposed by Greenland and Longnecker [14] and Orsini et al [15] to estimate the ln RR for one unit increase in ln CRP or ln IL-6. These methods are based on assuming a normal distribution of the log-transformed exposure and deal with the unbounded lowest and highest exposure categories and correlations between the effect estimates relating to the same reference group.

The association of circulating CRP with lung cancer risk was also assessed by sex. We also assessed the influence on our findings of study location (Europe vs. other populations), study design (cohort vs. nested case-control study), sample size (≥100 vs. <100 lung cancer cases), and CRP assay methodology [enzyme-linked immunosorbent assay (ELISA) vs. other assay].

Q and I^2^ statistics were used to examine whether the results of studies were homogeneous [16]. To avoid type II errors due to low power, the significance level was set at 0.10 instead of the more traditional 0.05 level. When statistical heterogeneity was detected, sensitivity analyses were performed. Publication bias was evaluated with Egger’s regression asymmetry test in which *P* value less than 0.10 was considered representative of statistically significant publication bias [17]. All statistical analyses were performed with Stata software, version 10 (Stata Corp, College Station, Texas). All statistical tests were two sided.

## Results

### Literature Search

**Figure 1:**
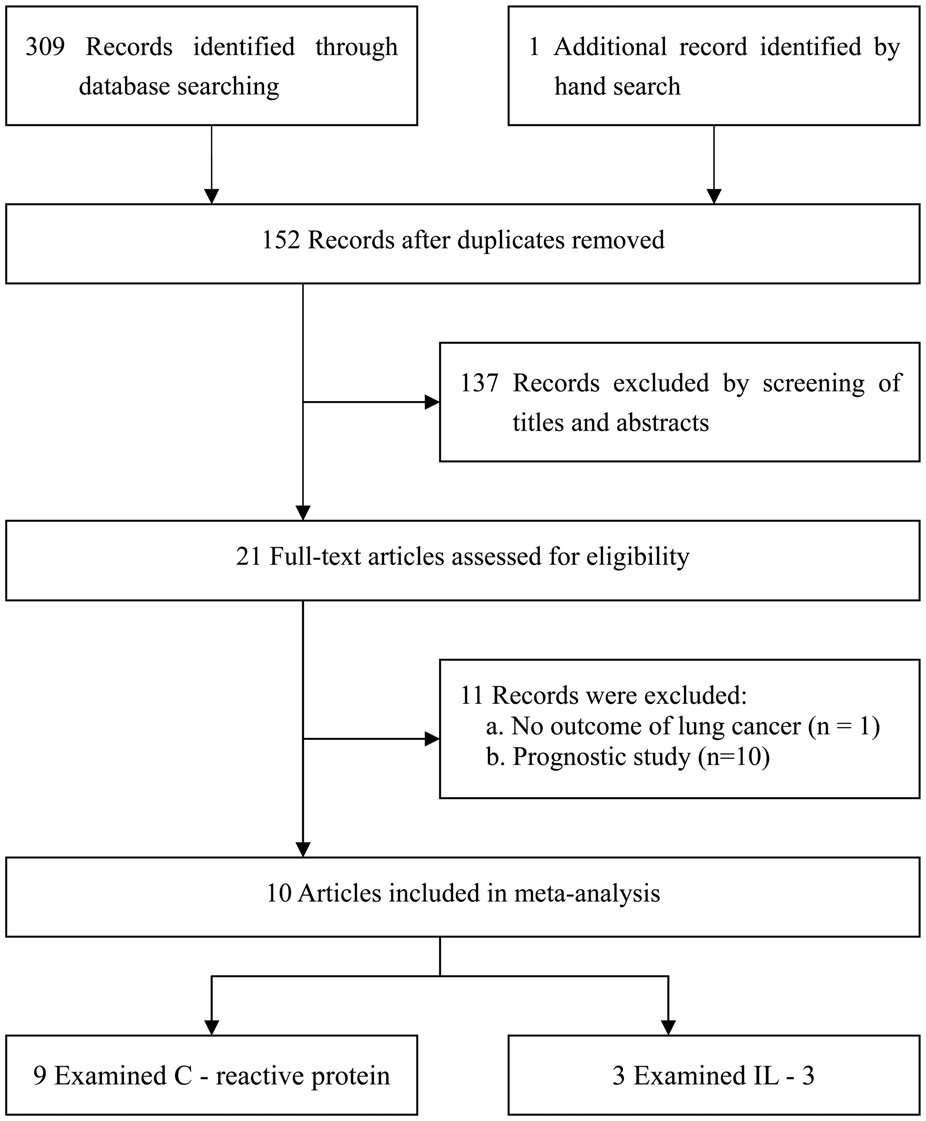
Flow diagram of study identification.

Our initial search strategy retrieved a total of 310 citations. After the titles and abstracts were screened, 289 articles were excluded because they were laboratory studies, review articles, or irrelevant to the current study. We identified 21 potentially relevant articles concerning CRP or IL-6 in relation to lung cancer risk. One publication was excluded because it investigated the association of CRP with risk of total cancer and there was no outcome of lung cancer [18]. Ten articles were excluded because they were prognostic study [19]–[28]. Finally, 9 articles concerning CRP (including 10 studies because 1 article [12] reported results from 2 independent cohorts) [12], [29]–[36] and 3 articles on IL-6 levels (including 5 studies because 1 article [12] reported results from 2 independent cohorts; and 1 article reported results from 2 independent case-control studies [37]) [12], [29], [37] were included in this meta-analysis (Figure 1). We performed this meta-analysis in accordance with the guidelines of the Preferred Reporting Items for Systematic Reviews and Meta-analyses (PRISMA) statement [38] (see checklist S1).

### CRP and Lung Cancer Risk

The 10 studies on CRP (7 cohort studies and 3 nested case-control studies) were published between 2005 and 2011 (Table 1) and involved a total of 1918 lung cancer cases. Seven studies were conducted in the Europe [12], [30], [32]–[34], [36], 2 in United States [29], [35], and 1 in Japan [31]. All studies determined circulating CRP concentration before cancer diagnosis as a single measurement in time. All studies used CRP assays were of high sensitivity except for the study by Van Hemelrijck [36]; 3 studies [29], [31], [34] used an ELISA methodology to measure high-sensitivity CRP, 3 used nephelometric assay [12], [33], 1 study used rate near-infrared particle immunoassay [32], 1 used an automatic latex agglutination photometric assay [30], and 1 used chemiluminescent immunoassay [35]. Most studies provided risk estimates that were adjusted for age (all 10 studies), smoking (8 studies) and body mass index (7 studies); fewer were adjusted for alcohol consumption (4 studies), and NSAID use (4 studies).

**Table 1:** Characteristics of studies on CRP and lung cancer risk.

First author	Year	Study	Country	Study design	Age, y	N. of participant	N. of Cases	Measure, mg/l	RR (95% CI)
Il’yasova (29)	2005	HABCS	US	Co	70–79	Total: 2438	42	ln CRP	1.64 (1.20–2.24)
Trichopoulos (30)	2006	EPICN	Greece	NCC	20–86	Control:996	72	1 SD of CRP*	1.31 (1.11–1.53)
Suzuki (31)	2006	JACC	Japan	NCC	40–79	Control: 425	209	<0.36	1.0
								0.36–0.81	1.13 (0.67–1.91)
								0.82–1.72	0.66 (0.38–1.16)
								>1.73	1.19 (0.70–2.02)
Siemes (32)	2006	Rotterdam	Netherlands	Co	≥55	Total: 6273	117	ln CRP	1.51 (1.21–1.88)
Allin (33)	2009	CCHS	Danish	Co	≥35	Total: 10121	255	<1	1.0
								1–3	1.5 (0.7–3.2)
								3–10	2.2 (1.0–4.6)
Heikkila (12)	2009	BWHHS	UK	Co	60–80	Total: 3274	23	ln CRP	1.03 (0.71–1.51)
Heikkila (12)	2009	CCS	UK	Co	45–59	Total: 1144	57	ln CRP	1.17 (0.91–1.50)
dos Santos Silva (34)	2010	NPHS-II	UK	Co	56.0†	Total:1868	35	0.037–1.340	1.0
								1.341–2.83	0.79 (0.24–2.62)
								2.84–6.38	1.18 (0.41–3.41)
								6.39–123.4	1.50 (0.55–4.08)
Chaturvedi (35)	2010	PLCO Trial	US	NCC	55–74	Control:670	592	<1.0	1.0
								1.1–2.7	1.22 (0.83–1.78)
								2.8–5.5	1.54 (1.08–2.21)
								>5.6	1.98 (1.35–2.89)
Van Hemelrijck (36)	2011	AMORIS	Sweden	Co	≥20	Total: 102749	516	Men<10	1.0
								10–15	1.34 (0.96–1.88)
								15–25	2.48 (1.46–4.19)
								25–50	2.02 (1.10–3.72)
								>50	1.38 (0.57–3.36)
								Women<10	1.0
								10–15	1.10 (0.76–1.60)
								15–25	1.99 (1.06–3.77)
								25–50	0.76 (0.24–2.38)
								>50	1.84 (0.76–4.48)

Abbreviation: RR, relative risk; CI, confidence intervals; CRP, C-reactive protein; NCC, nested case-control; Co, cohort; HABCS, Health Aging and Body Composition study; EPICN, European Prospective Investigation into Cancer and Nutrition; JACC, Japan Collaborative Cohort Study; CCHS, Copenhagen City Heart Study; BWHHS, British Women’s Heart and Health Study; CCS, Caerphilly Cohort Study; NPHS-II, Second Northwick Park Heart; PLCO, prospective Prostate, Lung, Colorectal, and Ovarian; AMORIS, Apolipoprotein MOrtality RISk.

† Mean.

* 1 SD = 3.2 mg/l.

**Figure 2:**
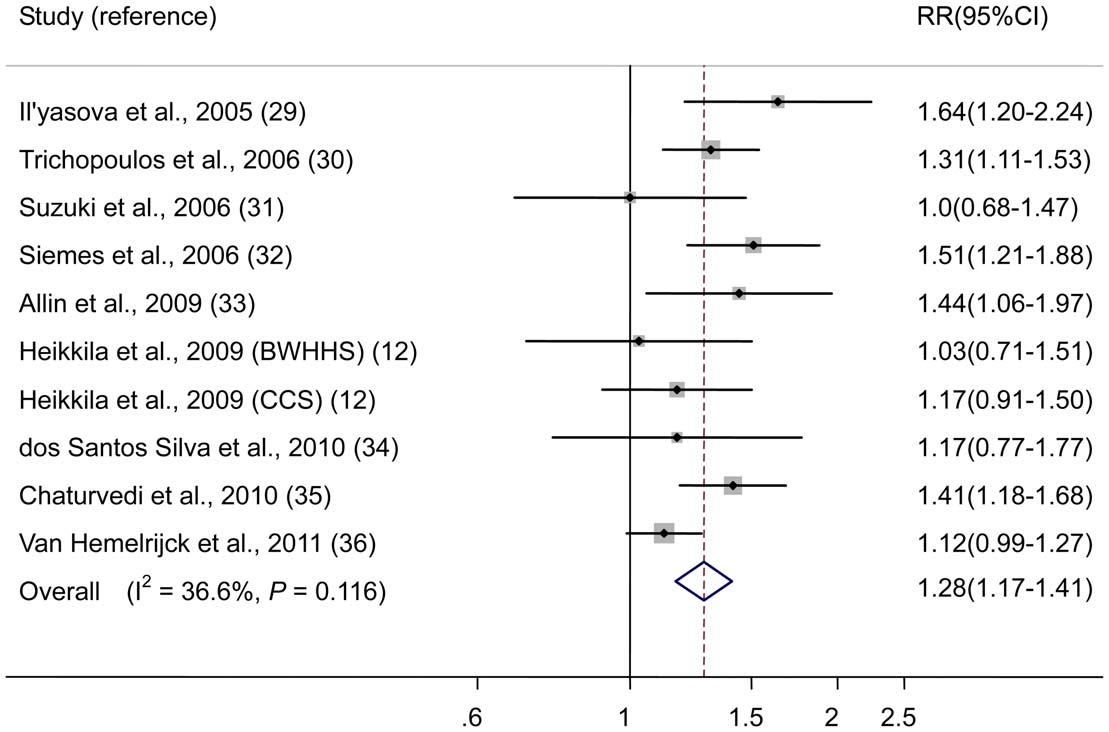
In studies on CRP, risk estimates of lung cancer associated with one unit change in ln CRP. Squares indicate study-specific risk estimates (size of the square reflects the study-specific statistical weight, i.e., the inverse of the variance); horizontal lines indicate 95% confidence intervals (CIs); diamonds indicate summary risk estimate with its corresponding 95% confidence interval. Abbreviation: BWHHS, British Women’s Heart and Health Study; CCS, Caerphilly Cohort Study.

The multivariable-adjusted RRs for each study and all studies combined for one unit change in ln CRP are shown in Figure 2. All studies on the association of CRP with lung cancer risk showed an positive association, which was statistically significant in 5 studies. The pooled RR of lung cancer for one unit change in ln CRP was 1.28 (95% CI 1.17–1.41). There was no statistically significant heterogeneity among studies (*P* = 0.116; I^2^ = 36.6%). The Egger test showed no evidence of publication bias (*P* = 0.750). After excluding the Van Hemelrijck et al. study [36] which did not use high-sensitivity CRP measurements, the association of high-sensitivity CRP with lung cancer risk was somewhat stronger (RR 1.33, 95% CI 1.23–1.45; *P* = 0.346, I^2^ = 10.6%).

In meta-analyses performed separately by sex, ln CRP was not associated with lung cancer in women, but a significant positive association was observed in men. The associations of ln CRP with lung cancer risk did not differ by study location, study type, sample size, and CRP assay methodology (Table 2).

**Table 2:** Summary risk estimates of the association between ln CRP and lung cancer risk.

Stratification group	References	RR (95% CI)			Heterogeneity test		
					*Q*	*P*	*I^2^* (%) †
All studies	12, 29–36	1.28	1.17–1.41		14.19	0.116	36.6
Gender							
Male	12,31,36	1.18	1.09–1.28		0.72	0.698	0
Female	12,31,36	1.05	0.95–1.15		0.09	0.957	0
Geographic region							
Europe	12,30,32–34,36	1.24		1.13–1.37	8.15	0.227	26.4
US	29,31,35	1.36		1.09–1.71	3.91	0.142	48.8
Study type							
Cohort	12,29,32–34,36	1.28		1.12–1.46	10.81	0.094	44.5
Nested case-control	30,31,35	1.31		1.15–1.50	2.54	0.281	21.2
Sample size							
<100	12,29,30,34	1.28		1.13–1.46	4.45	0.348	10.2
≥100	31–33,35,36	1.29		1.11–1.50	9.48	0.046	58.7
CRP assay methodology							
ELISA	29,31,34	1.27		0.93–1.72	4.16	0.125	51.9
Other assay	12,30,32,33,35,36	1.28		1.16–1.41	9.96	0.126	39.8

Abbreviation: RR, relative risk; CI, confidence intervals; ELISA, enzyme-linked immunosorbent assay.

† *I^2^* is interpreted as the proportion of total variation across studies that are due to heterogeneity rather than chance.

### IL-6 and Lung Cancer Risk

**Figure 3:**
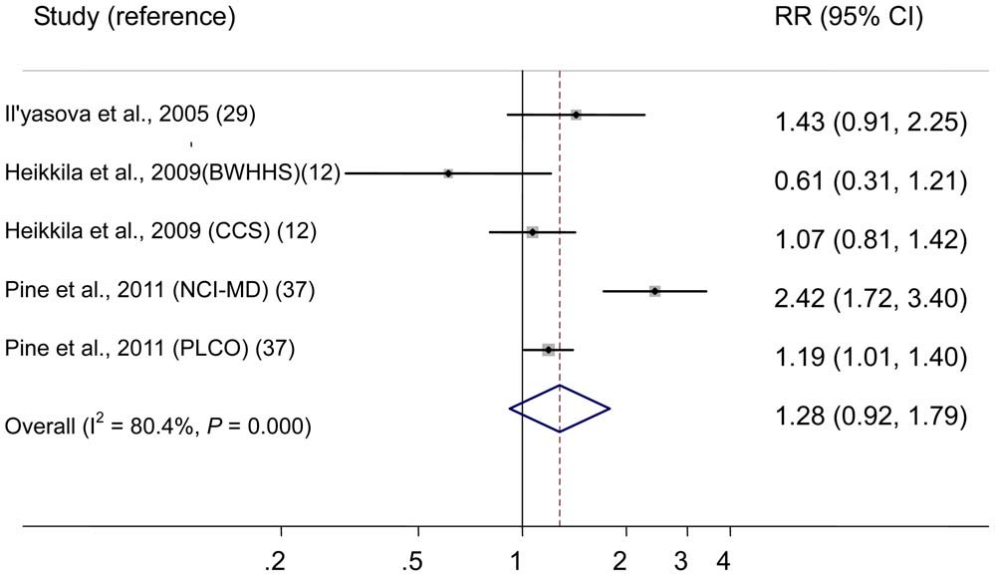
In studies on IL-6, risk estimates of lung cancer associated with one unit change in ln IL-6. Squares indicate study-specific risk estimates (size of the square reflects the study-specific statistical weight, i.e., the inverse of the variance); horizontal lines indicate 95% confidence intervals (CIs); diamonds indicate summary risk estimate with its corresponding 95% confidence interval. Abbreviation: BWHHS, British Women’s Heart and Health Study; CCS, Caerphilly Cohort Study; NCI-MD, National Cancer Institute-Maryland; PLCO, prospective Prostate, Lung, Colorectal, and Ovarian.

The 5 studies on IL-6 (3 cohort, 1 nested case-control, and 1 case-control study) were published between 2005 and 2011 (Table 3) and involved a total of 924 lung cancer cases. Three studies were conducted in the Unite States [29], [37], and 2 in Unite Kingdom [12]. The multivariable-adjusted RRs for each study and all studies combined for one unit change in ln IL-6 are shown in Figure 3. Results from studies on IL-6 in relation to lung cancer risk were inconsistent, with both inverse and positive associations reported. The pooled RR of lung cancer for one unit change in ln IL-6 was 1.28 (95% CI 0.92–1.79). There was statistically significant heterogeneity among studies (*P*<0.001, I^2^ = 80.4%). The Egger test showed no evidence of publication bias for CRP (*P* = 0.955).

**Table 3:** Characteristics of studies on IL-6 and lung cancer risk.

First author	Year	Study	Country	Study design	Age, y	N. of participant	N. of Cases	Measure, pg/ml	RR (95% CI)
Il’yasova (29)	2005	HABCS	US	Co	70–79	Total: 2438	42	ln IL-6	1.43 (0.91–2.26)
Heikkila (12)	2009	BWHHS	UK	Co	60–80	Total: 3274	23	ln IL-6	0.61 (0.31–1.22)
Heikkila (12)	2009	CCS	UK	Co	45–59	Total: 1144	57	ln IL-6	1.07 (0.81–1.43)
Pine (37)	2011	NCI-MD	US	CC	66.6†	Control:296	70	<1.4	1.0
								1.4–2.1	0.98 (0.51–1.86)
								2.1–3.8	2.28 (1.29–4.06)
								>3.8	3.29 (1.88–5.77)
Pine (37)	2011	PLCO Trial	US	NCC	55–74	Control:595	532	<2.7	1.0
								2.7–4.0	1.14 (0.79–1.65)
								4.0–6.6	1.25 (0.88–1.78)
								>6.6	1.48 (1.04–2.10)

Abbreviation: RR, relative risk; CI, confidence intervals; IL-6, Interleukin 6; NCC, nested case-control; Co, cohort; HABCS, Health Aging and Body Composition study; BWHHS, British Women’s Heart and Health Study; CCS, Caerphilly Cohort Study; NCI-MD, National Cancer Institute-Maryland; PLCO, prospective Prostate, Lung, Colorectal, and Ovarian.

To explore the heterogeneity among studies of IL-6 and lung cancer, we performed sensitivity analyses. By using a stepwise process, we determined that most of heterogeneity was accounted for the National Cancer Institute-Maryland (NCI-MD) study by Pine et al [37]. After excluding this single study, there was no study heterogeneity (*P* = 0.21, I^2^ = 34.6%), and the RR for one unit change in ln IL-6 was 1.13 (95% CI 0.93–1.38).

## Discussion

Our meta-analysis has assessed the relation between CRP, IL-6 and lung cancer risk. Overall, we identified a moderate positive association between pre-diagnostic CRP concentrations and lung cancer risk. And we found no clear support for an overall relationship between IL-6 and lung cancer risk.

Our summary estimate of CRP and lung cancer risk were similar to that of another recent meta-analysis [12]. This meta-analysis which included 6 studies with only 551 cases reported that a unit increase in ln CRP was associated with 32% increase in lung cancer risk. However, considerable heterogeneity was found (I^2^>70%). In contrast to that study, our meta-analysis involved a total of 1918 lung cancer cases and the summary risk estimate did not show any evidence of heterogeneity (I^2^ = 36.6%).

The biologic underlying mechanisms of the relationship between CRP levels and increased cancer risk are not yet definite. One hypothesis states that elevated levels of CRP are a marker of an underlying cancer or a premalignant state. Tumor growth can cause tissue inflammation around the tumor and hence increase plasma levels of CRP. Moreover, it is also possible that CRP is a part of the immune response of host which is studied as a consequence of tumor growth itself [39]. However, in most studies, CRP levels were measured well before development of cancer, so that associations were seen several years after CRP measurement, ruling out this causality.

Furthermore, smoking can not entirely explain this positive association, since most studies adjust for smoking. The study by Chaturvedi et al [35] also found that CRP levels were elevated among former smokers and were associated with increased lung cancer risk even among those who had quit smoking for up to 15 years. Nonetheless, evidences have indicated that cigarette smoke by itself can also induce pulmonary inflammation [40]. And Chaturvedi et al also found that high CRP levels among current smokers in relation to the amount smoked [35], which support the notion of a role of inflammatory pathways in tobacco-related lung cancer. In addition to smoking, several factors could also contribute to chronic pulmonary inflammation, including chronic lung infections with microorganisms such as mycobacteria or *Chlamydia pneumoniae*; and lung conditions such as asthma or pulmonary scarring. Indeed, all of these factors have been associated with increased lung cancer risk [41], [42].

There was some evidence that the association was stronger in men than in women. It has been well known that sex hormones play a role in the inflammation. Inflammation response might be differences between males and females. However, we still cannot draw the firm conclusion based on the limited published information and relatively small number of cases. Therefore, more studies with large sample are needed to further clarify these associations.

Lung cancer is classified into two major histological types, small cell lung carcinomas and non-small cell lung carcinomas [43]. Most investigations included modest numbers of lung cancers which precluding a precise estimation of risk among subgroups defined by lung cancer subtype. Only two studies considered the association of CRP with different histological subtypes [32], [35]. In the Rotterdam Study [32], CRP was associated with a increased risk of lung squamous cell carcinoma, but not adenocarcinoma. However, the number of lung adenocarcinoma (16 cases of 6273) in this cohort study was lower than lung squamous cell carcinoma (31 cases of 6273). In the nested case-control study by Chaturvedi et al [35], elevated CRP levels were significantly associated with risk of lung squamous cell carcinoma and small-cell cancer but not adenocarcinoma. And in this study, the number of lung adenocarcinoma (n = 269) was higher than lung squamous cell carcinoma (n = 126) which arguing against differences in statistical power as a potential explanation for the null association with adenocarcinoma. However, the reasons for these differential associations of CRP levels across lung cancer histologies are unclear and warrant further investigation.

In our meta-analysis, no association was found between IL-6 and lung cancer risk. The article by Pine et al reported significant positive associations of IL-6 and lung cancer risk [37]. This article included two independently studies which were NCI-MD study and Prostate, Lung, Colorectal, and Ovarian (PLCO) study. However, this article also showed that IL-6 levels were increased only among those with diagnosed lung cancer (NCI-MD study) or those who soon developed lung cancer (<2 years in PLCO study), whereas no association was seen at longer intervals (>2 years) in the PLCO study. Therefore, it is possible that IL-6 might participates primarily in tumor progression. This is supported by the evidence that increased circulating IL-6 levels are associated with lung cancer survival [44], and some reports that IL-6 is associated with tumor progression in several cancer types [45], [46].

The present study has several strengths. First, this latest meta-analysis combined all relevant literature published up to October of 2011. Moreover, meta-analysis of studies with large numbers of incident cases provides high statistical power for estimating the relationship between exposure and outcome risk. In addition, we normalized the variation between studies in the difference in exposure categories and further assessed dose response rather than simply categorical comparisons. Nevertheless, our meta-analysis has several limitations. First, all studies we included are observational. Observational studies, even when well controlled, are susceptible to various biases. Prospective cohort studies are less susceptible to bias than case-control studies because, in the prospective design, information on exposures is collected before the diagnosis of the disease. The positive association between CRP and lung cancer risk is supported by the prospective design of the 10 studies. Second, a meta-analysis is not able to solve problems with confounding factors that could be inherent in the included studies. Inadequate control for confounders may bias the results in either direction, toward exaggeration or underestimation of risk estimates. However, most studies in this meta-analysis adjusted for other known and potential risk factors for lung cancer. Third, heterogeneity may be introduced because of methodologic and demographic differences among studies. We used appropriate well-motivated inclusion criteria to maximize homogeneity, and performed sensitivity and subgroup analyses to investigate potential sources of heterogeneity. In this meta-analysis, the summary risk estimate of CRP did not show any evidence of heterogeneity. Finally, inherent in any review process of published studies is the possibility of publication bias. In this meta-analysis, we found no evidence of substantial publication bias.

In summary, findings from this meta-analysis indicated that CRP was associated with increased risk of lung cancer, especially among men. There was no significant association between IL-6 and lung cancer risk. More work is needed to further investigate the associations of CRP across lung cancer histologies.

## Supporting Information

Checklist S1 PRISMA Checklist for the meta-analysis.
(DOC)
